# Understanding the Variability of Associations Between Higher Ultra-Processed Food Consumption and Self-Reported Depression Severity: A Systematic Review of Epidemiological Studies

**DOI:** 10.3390/healthcare13182270

**Published:** 2025-09-11

**Authors:** Ume Odum, Mark Schure

**Affiliations:** Department of Human Development and Community Health, Montana State University, Bozeman, MT 59717, USA; mark.schure@montana.edu

**Keywords:** depression, ultra-processed food consumption, unhealthy eating, health behavior, Nova food classification system

## Abstract

**Background/Objectives**: Coinciding with an increasing trend of ultra-processed food (UPF) consumption, nations are experiencing a surge of diet-related chronic diseases such as diabetes and other metabolic conditions. While many studies have found positive associations of UPF consumption with physical health outcomes, few studies have examined the effects of UPF consumption on mental health conditions. Therefore, the purpose of this systematic review is to understand the variability of associations between higher UPF consumption and depression severity across research studies with adult populations. **Methods**: Using PsychINFO, CINAHL Ultimate and Web of Science databases, we conducted a systematic review of recent observational studies examining the association of UPF consumption with self-reported depression in adult populations. To assess the quality of each study, Strengthening the Reporting of Observational Studies in Epidemiology—Modified (STROBE-M) checklist was used to assess risk of bias in each of the included studies. **Results**: Of the 530 records retrieved, 13 studies with a total of 264,519 participants met all eligibility criteria and were included in this review. All 13 studies reported a positive association between higher UPF consumption and depression severity, ranging from 1.15–2.04 (Hazard, Odds, and Relative Risk ratios). Some of the studies likely experienced selection bias due to loss of participants to follow-up. **Conclusions**: In adult populations, observational studies indicated a low to moderately increased risk of self-reported depression among those with diets that include higher amounts of ultra-processed foods.

## 1. Introduction

Growing evidence from research on ultra-processed food (UPF) consumption and its negative health outcomes [1,2] suggests an emerging diet-related disease burden. According to Nova (the most widely used food classification system), food items can be classified into four distinct groups based on their level of processing: (1) minimally processed or unprocessed foods, (2) processed culinary ingredients, (3) processed foods, and (4) ultra-processed foods [3]. UPFs are industrial formulations of ingredients from oils, fats, sugar, starch, proteins, and organic substances—additives (some used in the production of cosmetics), colors, flavors, sweeteners, and emulsifiers [4,5]. Examples of UPFs include ice creams, frozen desserts, margarines, spreads, soft drinks, sweetened or salty packaged snacks, confectionery, packaged breads, reconstituted meat products and pre-prepared frozen or shelf-stable dishes [6,7]. They are produced for long shelf life and are enticing and addictive to many consumers [4,8,9]. They are made through molding, extrusion and industrial frying [6].

UPFs are easily marketed to consumers due to their relative affordability and convenience [10]. However, emerging evidence suggests that increased UPF consumption comes with personal health consequences. For example, the excessive consumption of UPFs has been found to be associated with cardiovascular diseases, cancer, obesity, diabetes and depression [2,11,12]. People who consume high amounts of UPFs are also more likely to consume less nutritious food types with protein, fiber, fruits, and vegetables [13,14]. Aside from UPFs being less nutritious, ultra-processing introduces glycation end products, which may lead to gut and metabolic diseases that have been linked to depression [15].

Depression is a common and serious medical illness that negatively affects how a person feels, thinks, and acts [16]. Depression may induce feelings of guilt and worthlessness, loss of interest in activities once enjoyed, and changes in eating and sleeping patterns [16]. It is a common mental health disorder that affects more women than men. Community studies have largely shown higher lifetime rates of depression among women compared to men. [17]. Depression is one of the five leading causes of years lived with disability, mostly in low- and middle-income countries [18]. Depression is the leading cause of disease burden globally, affecting more than 320 million people [18].

To the best of our knowledge, no prior systematic reviews have been conducted to understand the variability in the magnitude of associations between UPF consumption and depression severity across studies among adult populations. Previous systematic reviews have only focused on the association between UPF consumption and depression or in combination with other mental health outcomes in adolescent and adult populations [19,20,21,22]. Therefore, the purpose of this systematic review is to understand the variability of associations between higher UPF consumption and depression severity across observational studies in adult populations. In this paper, we present: (1) a detailed process for article selection and a summary of data extraction and quality assessment for each selected study, (2) a synthesis of study results regarding the quantitative associations of UPF consumption with depression among adult populations, and (3) a discussion and critique of the systematic review findings.

## 2. Materials and Methods

This review seeks to answer the following question: What is the variability in the magnitude of associations between higher UPF consumption and depression severity across studies with different adult populations? This systematic review is conducted in accordance with the Preferred Reporting Items for Systematic reviews and Meta-Analyses (PRISMA) 2020 guidelines [23]. We followed the PRISMA 2020 guidelines to ensure transparency regarding our engagement in this systematic review, including what we did and what we found [23]. This review protocol was developed and registered (registration number INPLASY202570071) with the International Platform of Registered Systematic Review and Meta-analysis Protocols (INPLASY).

### 2.1. Eligibility Criteria

For a study to be included in this review, each observational study had to identify UPF consumption as the exposure and depression (including depressive symptoms) and risk of depression as the outcome. In addition, studies needed to: (1) include only adult participants (18 years and older), (2) be peer-reviewed, and (3) be published in English from 2014 or later so as not to replicate earlier similar reviews. We chose to focus on adult populations only, as earlier systematic reviews included adolescent populations. We excluded reviews, abstract-only papers, experimental studies, letters, editorials, and studies that were not published in English.

### 2.2. Information Sources and Search Strategies

To ensure no relevant articles were left out, an extensive and in-depth search strategy was last carried out in October 2024 using PsycINFO, CINAHL Ultimate, and Web of Science databases. They are: “highly processed” or ultra-processed AND food* AND depression. Ultra-processed OR “ultra processed” AND food* AND depression NOT diabet* OR cardio* OR hypertension OR obes* OR cancer OR carcinoma. “soft” OR “soda” OR “confectionary” OR “sweet” OR “sugary” OR “high fat” OR “most processed” AND “food*” OR “drink*” OR “beverage*” OR “product*” OR “good*” AND “risk of depression” OR “depressive disorder*” OR “major depressive disorder” OR “depression” OR “unipolar depression” OR “bipolar depression” OR “depressive symptoms”. A hand search of articles’ reference lists was also conducted.

### 2.3. Data Extraction

We used a structured form to collect and summarize relevant data from eligible studies. Some of the data extracted include: first author surname, year of publication, study design, study population, sample size, country of study, exposure and outcome assessment tools, and respective associations/effect sizes (Hazard Ratios [HRs], Odds Ratios [ORs], and Risk Ratios [RRs]) with 95% Confidence Intervals [CIs]. This was independently done by both authors. Data was collected by the first author, while the second author confirmed the data. Differences were resolved through unanimous consensus or referencing group four of the Nova food classification system for clarity. When there was a lack of clarity on study design, each author discussed the rationale for their decision based on the information provided in each publication about how and when the study data were collected.

### 2.4. Quality Assessment

Risk of bias assessment was evaluated by both authors. Differences were resolved by consensus. We used the Strengthening the Reporting of Observational Studies in Epidemiology—Modified (STROBE-M) checklist [24] to assess the quality of the included studies. Risk of bias for each of the eligible studies was evaluated on a scoring scale that was made up of 70 items for both the case-control and cohort studies and 63 for the cross-sectional studies. Depending on the item, it was decided to have scores of 0 if the checklist item is not fulfilled, a score of 1, 2, or 3 if the checklist item is fulfilled, and a score of NA if the checklist item is not applicable for the included study. This was done to determine the overall quality score for each of the included studies. Each of the studies included was categorized as either excellent (≥85), good (70 to <85), fair (50 to <70), or poor (<50) quality based on the overall scores in percentage.

### 2.5. Data Synthesis

Due to the minimal number of included studies and variability of measurements in both the exposure and outcome—which may not provide a meaningful and reliable analysis that would be robust or lead to generalizable conclusions—we did not conduct a meta-analysis. To systematically synthesize findings from the included studies, we did a narrative synthesis. We tabulated study findings and characteristics. This was done to provide useful information for the association of interest.

## 3. Results

### 3.1. Selection Process

**Figure 1:**
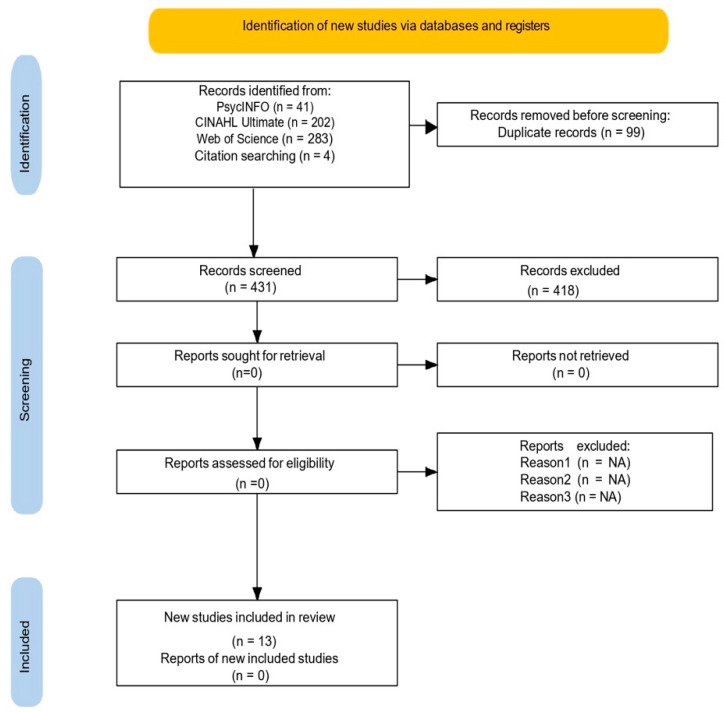
PRISMA diagram [25] for the search protocol and the inclusion and exclusion of the reviewed articles. No automation tools were used for the exclusion process; records were excluded manually by the first author. “Citation searching” refers to the hand search of the included studies’ reference lists.

Figure 1 provides a summary of the selection process. To answer the review question, we identified 530 study records from PsycINFO, CINAHL Ultimate, and Web of Science databases with a hand search of the included studies’ reference lists, which last took place in October 2024. After the removal of 99 duplicate records, 431 of the 530 study records were left for screening based on the listed eligibility requirements. Two reviewers independently screened the abstracts and studies to determine study eligibility. After the screening process, 418 records were removed (417 records were removed based on inclusion/exclusion criteria, and one record was different from the association of interest, i.e., depression was the exposure and UPF consumption the outcome). Finally, 13 studies (eight cohort, one case-study and four cross-sectional) met all eligibility criteria and were included in this review.

### 3.2. Study Characteristics

Table 1 contains a summary of study results. From the included studies, the study participant sample size ranged from 596 to 263,923. Each study differed across demographics. For example, one study [26] focused on college students, another on older adults [27], and in different countries. China [28], Brazil [29], Spain [26], Italy [30], Australia [13], France [31], and Korea [32] each had one study. The United States of America (USA) [27,33], the United Kingdom (UK) [34,35], and Japan [36,37] each had two studies. Most of the studies [13,26,27,28,29,30,34] used the Food Frequency Questionnaire (FFQ) to assess the exposure and the Center for Epidemiologic Studies Depression Scale (CES-D) to assess the outcome [30,31,34,36,37]. All 13 studies reported a positive association between higher ultra-processed food consumption and depression severity, with HRs, ORs and RRs ranging from 1.15–2.04, suggesting an overall strong association between higher UPF consumption and depression severity.

Overall, the studies show a statistically significant association between higher UPF consumption and depression severity. There were differences in UPF consumption across marital status. For example, most of the studies reported more consumption among those not married than their married counterparts. For depression, studies differed, with females reporting greater depression than males. Confounding factors such as sex, physical activity (PA) levels and health conditions were reported and adjusted in all the included studies. For example, even after adjusting for physical activity (PA) levels, Lee and Choi [32] still found a higher UPF consumption and depression severity among those not engaging in PA than those who were actively engaging in PA.

Each study used the Nova food classification system in the identification of UPF consumption. For what constituted the exposure, all studies differed. One assessed sugar intake [35], confectionery intake [37], sweetened beverages [27], two assessed soft drinks consumption [28,36], and eight [13,26,29,30,31,32,33,34] assessed the entire food items in group four of the Nova food classification system. The reviewed studies also differed in the depression outcome. One assessed depression [32], depression risk [27], elevated psychological distress [13], three assessed incidences of depression [26,29,35] and seven [28,30,31,33,34,36,37] assessed depressive symptoms.

### 3.3. Quality Assessment Outcomes

For the quality assessment (See last column in Table 1), only one cohort study was rated a lower quality score of 77%, while the other cohort studies’ quality scores ranged from 87–91%. The cross-sectional studies’ quality scores ranged from 75–84%. There were consistent absences of information provided in the respective studies. Only one study [35], provided a clear hypothesis of the direction of association between the exposure and outcome. Only two studies [13,26], defined and discussed effect modifiers. Six of the thirteen studies addressed ways to minimize sources of potential bias [13,26,27,31,34,36]. Only one [37] discussed the required analytical power to compute statistics. Seven studies discussed methods for handling missing data [13,27,31,34,35,36,37]. Lastly, only three out of the eight cohort studies discussed how loss-to-follow-up was addressed [26,31,34].

## 4. Discussion

### 4.1. Summary of Findings

This systematic study sought to determine the variability of association among observational studies examining the quantitative relationship between UPF consumption and depression severity in adult populations. All included studies reported a low to moderate positive association between higher UPF consumption and self-reported depression. Although with different tools of assessment for both the exposure and outcome across the included studies, study findings show that higher UPF consumption is associated with self-reported depression severity. All studies grouped the level of UPF consumption into categories, with the highest group serving as the exposure group of concern.

The magnitude of association between UPF consumption and depression for studies included in this systematic review is comparable to that found in other reviews, with most relative risk measures ranging between 1 and 2 [21,22]. As both reviews included studies with adolescent populations, it is difficult to draw direct comparisons with this review, which is focused on adult populations. Furthermore, compared to this review, the other reviews were mainly comprised of cross-sectional studies. Therefore, the ability to determine causality remains a limitation of existing reviews on this topic.

### 4.2. Implications and Future Directions

This review builds upon earlier reviews and observational studies examining the strength of association between UPF consumption and depression. The current evidence shows a significant positive relationship between a diet comprised of greater amounts of UPFs and self-reported depression severity. Furthermore, the magnitude of associations is mostly consistent across studies with diverse geographic and demographic populations. This would suggest that, despite potential inherent biases within each of these studies, there is sufficient reason to warrant further studies that can rigorously examine causal pathways of UPF consumption with depression and other relevant mental and behavioral health outcomes.

It is important to point out at this time that the relationship between UPF consumption and depression may not be linear. A variety of hypotheses have emerged and been summarized that provide direction for future mechanistic research studies [10]. Carefully designed longitudinal studies will be required to provide further clarity on risk and protective factors that may confound the UPF–depression relationship. These, in turn, will provide directions to practitioners on promising new interventions and therapies that may lead to improved health outcomes.

Considering the modern-day reality of our food industry, researchers can shape our understanding of the risks of adopting diets that are designated as ultra processed. Researchers-turned advocates will be important to direct policy and programming that informs the public about the links between a healthy diet and better health outcomes, and leads to better consumer decision-making with this knowledge. This is not to say that “food addictions” can be easily addressed. To adequately respond to the real challenges posed by the food industry, deliberate actions will need to include the expertise of practitioners, the influence of policymakers, and the political will of the people.

### 4.3. Study Limitations

This systematic review and its findings should be interpreted considering several important limitations. First, several of the included cohort studies were vulnerable to selection bias due to loss to follow-up. Participants who dropped out may have been systematically different from those who remained (e.g., individuals with worsening depression may stop responding to follow-up dietary surveys, leaving only healthier individuals). Such attrition could result in biased estimates and underestimation of the true association between UPF consumption and depression. Similar concerns have been raised in large-scale cohort studies such as the European Prospective Investigation into Cancer and Nutrition (EPIC), where attrition substantially affected exposure–outcome relationships [38]. Second, reliance on self-reported dietary intake measures (e.g., FFQ and 24 h DR) may have introduced recall bias (participants may not accurately remember or report their food intake). For example, participants with depression may underreport their UPF consumption, which could distort the measured association. This could lead to participants being misclassified. Third, although most studies adjusted for known confounders such as sex, physical activity, and baseline health conditions, they remained at risk of residual confounding. Important factors such as genetic predisposition to depression, socioeconomic status, and cultural dietary patterns were not consistently controlled. Evidence from twin studies suggests that genetic risk alone accounts for up to 40% of the variance in depression outcomes, underscoring the potential magnitude of omitted confounders [39]. For example, genetic predisposition to depression could explain part of the association between UPF and depression, but such data are often unavailable. Fourth, the possibility of reverse causality cannot be ruled out. While the included studies assumed that UPF consumption preceded depression, depression itself may lead to increased UPF intake through mechanisms such as comfort eating or reduced motivation to prepare fresh meals. The Nurses’ Health Study, for example, demonstrated that depressive symptoms predicted subsequent changes in dietary patterns, suggesting bidirectional associations that complicate causal inference.

The generalizability of findings from included studies is limited. All included studies were conducted in economically developed countries, such as Japan, the United States, and those in Europe. Two studies sampled only Japanese workers, while another focused exclusively on older U.S. adults. These narrow populations limit external validity, as dietary patterns and determinants of depression differ in low- and middle-income countries (LMICs). Findings from economically advantaged settings may therefore not translate to regions where food systems and socioeconomic stressors differ markedly.

Another critique to consider involves the review’s inclusion and exclusion criteria. Restricting to peer-reviewed studies published in English likely excluded relevant work in other languages, particularly from non-Western contexts. Similarly, by excluding non-adult populations, we overlooked evidence in children and adolescents, groups that consume disproportionately high levels of UPF. Our reliance on only three major databases also raises the possibility of publication bias, as gray literature and regional databases were not included. Finally, our restriction on studies published between 2014 and later, while intended to capture recent evidence, may have excluded earlier but still relevant studies. For example, a strong study before 2014 linking UPF to depression was not considered. Additionally, studies published after our last search date (October 2024) were necessarily missed, thereby introducing time-lag bias.

### 4.4. Conclusions

This review of recent observational studies shows a relatively consistent positive association of UPF consumption with self-reported depression severity among adult populations across different geographic regions. An increasing number of longitudinal studies bring the field closer to establishing a causal direction whereby greater UPF consumption leads to a greater risk of depression. Further rigorously designed studies are needed to tease out potential risk and protective factors that may lead to biased findings.

## 5. Registration and Protocol

This review protocol was developed and registered (registration number INPLASY202570071) with INPLASY.

## Figures and Tables

**Table 1:** Summary of Reviewed Studies.

First Author Last Name (Year)	Study Type	Exposure Assessment	Outcome Assessment	Age	Size	Association	Dose-Response	Measure of Association (95%)	Quality Score
Yu (2015) [28]	CSS	FFQ	SDS	≥18	3667	Yes	Yes	OR: 2.00 (1.15, 3.37)	Good
Leal (2023) [29]	CS	FFQ	SRQ	≥18	2572	Yes	Yes	HR: 1.82 (1.15, 2.88)	Excellent
Kashino (2021) [36]	CS	DHQ	CES-D	≥18	935	Yes	Yes	OR: 1.91 (1.11, 3.29)	Excellent
Gomez-Donoso (2020) [26]	CS	FFQ	SRMDD/ADU	≥18	14,907	Yes	Yes	HR: 1.33 (1.07, 1.64)	Excellent
Zheng (2020) [33]	CSS	24 h DR	PHQ	≥20	13,637	Yes	Yes	OR: 1.34 (1.00, 1.78)	Good
Godos (2023) [30]	CSS	FFQ	CES-D	≥18	596	Yes	Yes	OR: 2.04 (1.04, 4.01)	Good
Arshad (2024) [34]	CS	FFQ	CES-D	≥18	4554	Yes	Yes	OR: 1.31 (1.04, 1.64)	Excellent
Kaiser (2023) [35]	CS	24 h DR	ICD-10 CODES	39–72	188,426	Yes	Yes	HR: 1.15 (1.07, 1.24)	Excellent
Lane (2023) [13]	CS	FFQ	K-10	27–79	23,299	Yes	Yes	OR: 1.23 (1.10, 1.38)	Excellent
Adjibade (2019) [31]	CS	24 h DR	CES-D	18–86	26,730	Yes	Yes	HR: 1.21 (1.15, 1.27)	Excellent
Lee (2023) [32]	CSS	24 h DR	PHQ	≥20	9463	Yes	Yes	OR: 1.40 (1.00, 1.96)	Good
Shimmura (2022) [37]	CS	DHQ	CES-D	19–68	911	Yes	Yes	OR: 1.72 (1.03, 2.86)	Excellent
Guo (2014) [27]	CC	FFQ	SRQ	50–71	263,923	Yes	Yes	OR: 1.30 (1.17, 1.44)	Good

Notes: CC—Case-control study, CSS—Cross-sectional study, FFQ—Food Frequency Questionnaire, SDS—Self rating depression scale, CS—Cohort study, DHQ—Diet history questionnaire, SRQ—Self report questionnaire, 24 h DR—24 h dietary recall interview/questionnaire, CES-D—Center for epidemiologic studies depression scale, SRMDD—Self-reported medically diagnosed depression, ADU—Antidepressant use, PHQ—Patient health questionnaire, ICD-10 codes—International statistical classification of diseases and related health problems, tenth revision codes (F32 and F33), K-10—Ten-item Kessler psychological distress.

## Data Availability

No new data were created or analyzed in this study.
